# Chemically Stressed Bacterial Communities in Anaerobic Digesters Exhibit Resilience and Ecological Flexibility

**DOI:** 10.3389/fmicb.2020.00867

**Published:** 2020-05-12

**Authors:** Benjamin Schwan, Christian Abendroth, Adriel Latorre-Pérez, Manuel Porcar, Cristina Vilanova, Christina Dornack

**Affiliations:** ^1^Institute of Waste Management and Circular Economy, Technische Universität Dresden, Pirna, Germany; ^2^Robert Boyle Institut e.V., Jena, Germany; ^3^Darwin Bioprospecting Excellence, S.L. Parc Cientific Universitat de València, Paterna, Spain; ^4^Institute for Integrative Systems Biology, University of Valencia-CSIC, Paterna, Spain

**Keywords:** anaerobic digestion, Lotka–Volterra, population modeling, anaerobic microbiomes, microbiome manipulation

## Abstract

Anaerobic digestion is a technology known for its potential in terms of methane production. During the digestion process, multiple metabolites of high value are synthesized. However, recent works have demonstrated the high robustness and resilience of the involved microbiomes; these attributes make it difficult to manipulate them in such a way that a specific metabolite is predominantly produced. Therefore, an exact understanding of the manipulability of anaerobic microbiomes may open up a treasure box for bio-based industries. In the present work, the effect of nalidixic acid, γ-aminobutyric acid (GABA), and sodium phosphate on the microbiome of digested sewage sludge from a water treatment plant fed with glucose was investigated. Despite of the induced process perturbations, high stability was observed at the phylum level. However, strong variations were observed at the genus level, especially for the genera *Trichococcus, Candidatus Caldatribacterium*, and *Phascolarctobacterium.* Ecological interactions were analyzed based on the Lotka–Volterra model for *Trichococcus*, *Rikenellaceae DMER64*, *Sedimentibacter*, *Candidatus Cloacimonas*, *Smithella, Cloacimonadaceae* W5 and *Longilinea*. These genera dynamically shifted among positive, negative or no correlation, depending on the applied stressor, which indicates a surprisingly dynamic behavior. Globally, the presented work suggests a massive resilience and stability of the methanogenic communities coupled with a surprising flexibility of the particular microbial key players involved in the process.

## Background

In previous decades, tremendous efforts have been made to better understand the biocenosis underlying the process of anaerobic digestion. According to a recent study, approximately 300 operational taxonomic units (OTUs) represent 80% of the microorganisms involved in anaerobic digester microbiomes. If the remaining 20% are also taken into consideration, the number of OTUs is much higher (Kirkegaard et al., 2017). Moreover, an often complex and inhomogeneous feedstock is used, which can affect microbial community structures and functions (Xu et al., 2018). To gain better access to microbial systems of such complexity, high-throughput approaches are often applied, such as 16S-rRNA gene amplicon sequencing (Abendroth et al., 2015), metagenomics (Xu et al., 2019); or metaproteomics (Hassa et al., 2018), all of which facilitate the analysis of complex microbial communities with high diversity. The continuously decreasing prices of these technologies have allowed scientists to compare many anaerobic digester plants simultaneously. For example, Sundberg et al. (2013) compared 21 full-scale anaerobic digesters, including co-digesters and sewage sludge digesters, based on 16S-rRNA amplicon sequencing at both mesophilic and thermophilic temperatures. In the study by Sundberg et al. (2013), Actinobacteria, Proteobacteria, Chloroflexi, Spirochetes and Euryarchaeota were dominant in sewage sludge digesters, while Firmicutes were especially enriched in co-digesters. Theuerl et al. (2015) indicated that even well-operating agricultural biogas plants show fluctuation in the microbial community composition due to high sensitivity to changes in the process performance.

The aforementioned studies provide good insight into microbial key players involved in the process of anaerobic digestion. However, to understand the reasons behind the observed taxonomic patterns, complex experiments are necessary, which usually involve disturbing the system to identify the changes associated with the new environment. The experiments reported include stressors like very low pH of 6.0 (Delbès et al., 2000; Hori et al., 2006; Abendroth et al., 2017), changing temperature (Shi et al., 2019), very high salt concentrations causing conductivity values up to 80 mS cm^–1^ (Ogata et al., 2016; De Vrieze et al., 2017) and varying total solids (TS) contents (Hardegen et al., 2018). For instance, to further test the hypothesis that the genus *Methanosarcina* is especially enriched in anaerobic digester sludge with low viscosity (Abendroth et al., 2015), an experiment was conducted in which sewage sludge was fed in parallel with various feedstocks with different percentages of TS (Hardegen et al., 2018). Hardegen et al. (2018) gradually increased the concentration of total volatile fatty acids (up to 10 g L^–1^ before acidosis took place); as the researchers anticipated, the approach in which a feedstock with a low percentage of TS was used resulted in higher concentrations of *Methanosarcina* than the approach with feedstocks with high concentrations of TS were fed did. In another example, Spirito et al. (2018) used antibiotics up to concentrations of 5 mg L^–1^ (monensins) to disturb the underlying microbiome. An adaptation to extremely high concentrations of monensins was possible, which was explained by the authors with a highly redundant microbiome, in which the inhibited species can be substituted by other microorganisms with similar functions.

Experiments with such harsh conditions-like those in the experiments performed by De Vrieze et al. (2017) and Spirito et al. (2018)-make it possible to study the microbial shifts caused by different stress levels; however, this provides no insight *per se* into the microbial interactions that are driving these shifts. With massive sequencing data, it would be possible to find biological correlations by, for example, pairwise comparisons or regression- and rule-based networks, enabling an approximate calculation of microbial interactions (Faust and Raes, 2012). According to Faust and Raes (2012), this would make it possible to determine whether positive, negative or neutral effects exist between different species, indicating potential ecological interactions, such as mutualism, commensalism, parasitism, amensalism or competition. Because of this, scientists are regularly trying to understand microbial interactions within anaerobic microbiomes through sequencing data. For example, Kuroda et al. (2016) analyzed the correlations between multiple OTUs within granules from an anaerobic upstream sludge blanket (UASB). In that work, many positive correlations between methanogens and syntrophic bacteria were highlighted. The existing microbial interaction between syntrophs and methanogens has been investigated since the 1980s (Baresi et al., 1978), and the work of Kuroda et al. (2016) highlighted the applicability of sequencing-based information on microbial ecology. In many more studies, based on sequencing approaches, to shed light on microbial interactions. Very often, network analysis is used to analyze the evolution of microbiomes based on 16S-rRNA gene amplicon sequencing in response to a certain environmental stress. For instance, a recently applied network analysis demonstrated that organic overloading causes microbial population shifts, which in turn affects microbial interactions (Braz et al., 2019).

Although several reports have investigated microbial interactions within anaerobic microbiomes, to date, it has not been determined whether interactions may be restricted to certain environmental conditions. For example, it is conceivable that two mutualistic bacteria shift into a state of parasitism due to changing digester conditions in which the feedstock composition changes. Using Lotka–Volterra based modeling, the presented work aims to address the question of how microorganisms in anaerobic microbiomes are ecologically adapting to externally induced fluctuations. To answer this question, four semicontinuously fed reactors were treated over 9 weeks while receiving different inhibiting substances, namely nalidixic acid, γ-aminobutyric acid (GABA) and sodium phosphate. Following this, 16S-rRNA gene amplicon sequencing and Lotka–Volterra modeling were applied to address the microbial interactions in all four reactors. Based on DNA sequencing, gLV has already been applied various times to investigate microbial interactions in the gut (Weng et al., 2017), in cheese (Mounier et al., 2008), in the coffee-machine bacteriome (Vilanova et al., 2015) and its suitability to simulate population dynamics and estimate microbial interactions based on high-throughput sequencing was recently highlighted by Kuntal et al. (2019).

## Materials and Methods

### Inoculum and Substrates

As seed sludge, a digester sludge from a sewage plant in Saxonia was used. The sludge came from the digestion towers of a large sewage treatment plant in Saxony, Germany. The average solids retention time (SRT) in the digestion towers is 16.5 days. Biogas is produced under mesophilic conditions in the range of 30–35°C. The average pH value is 7.7. The TS content varies between 3 and 5 g L^–1^ per year. The sum of the volatile fatty acids (VFA) amounts to 163 mg L^–1^ on average. At the time of sampling, this sum parameter was 169 mg L^–1^. The ammonium content was 1157 mg L^–1^.

**FIGURE 1:**
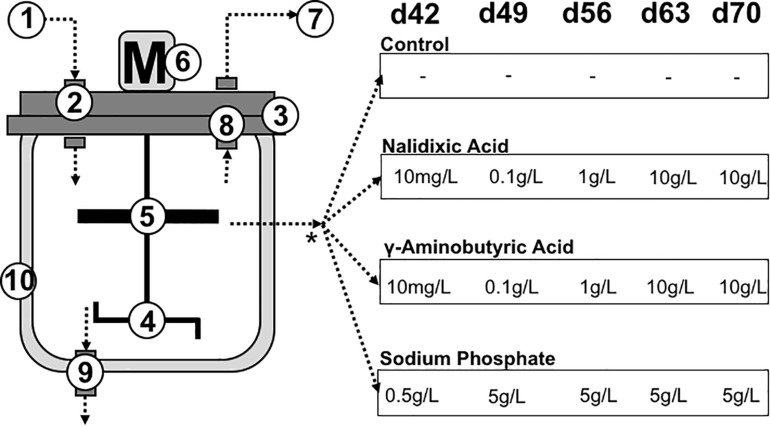
Reactor set-up: To compare the influence of different inhibitors on microbial interactions, four experiments were performed in continuous stirred tank reactors. The left side of the figure shows the reactor design: **(1)** Substrate input, **(2)** gas tight feeding pipe, **(3)** lid, **(4)** stirrer, **(5)** upper stirrer for foam elimination, **(6)** motor for the stirrer, **(7)** gas output, **(8)** gas tight pipe, **(9)** sampling port, **(10)** double wall for heating. The experiment lasted 77 days, with chemical inhibitors applied on day 42, 49, 56, 63, and 70. Nalidixic acid, γ-aminobutyric acid (GABA) and sodium phosphate were applied as chemical inhibitors. One reactor received no additional inhibiting chemicals to function as a control.

The reactors were supplemented with nalidixic acid (Sigma Aldrich, Germany), GABA (Sigma Aldrich) or sodium phosphate (Sigma, Aldrich), which were applied as stressors during the last 5 weeks, as shown in Figure 1. To prevent starvation, glucose was used as substrate.

### Reactor Performance

**FIGURE 2:**
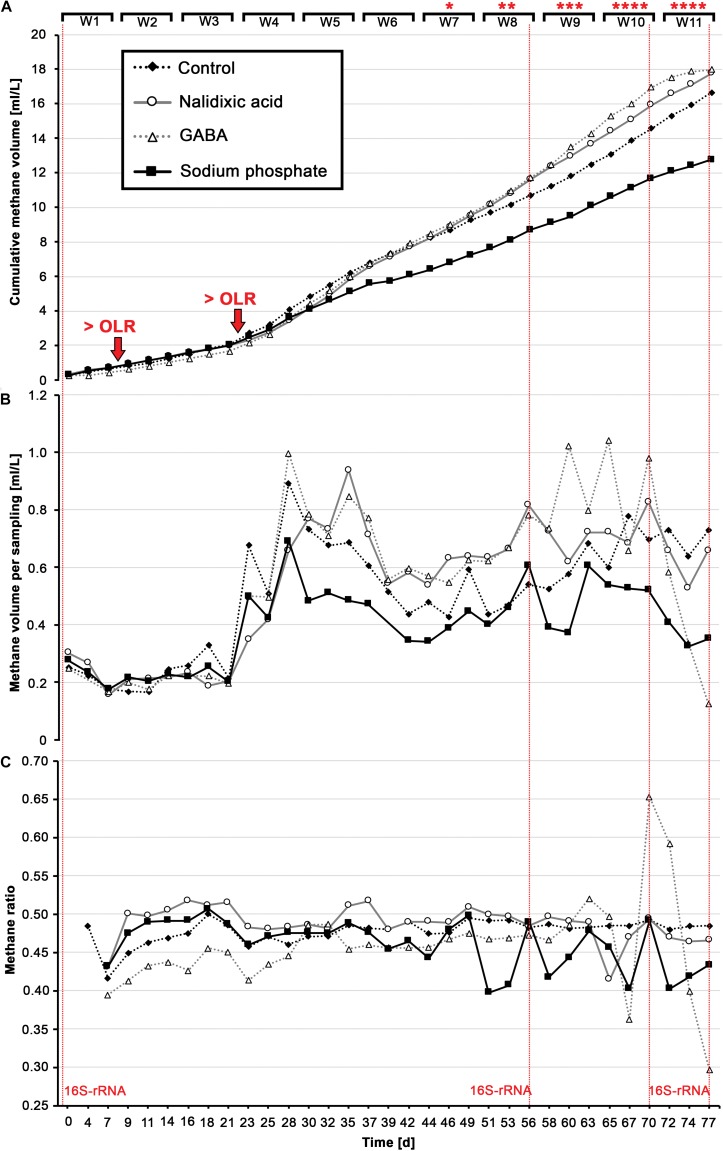
Produced biogas: Cumulative methane **(A)**, the amount of methane per sampling day **(B)** and the ratio of methane to total biogas for each sampling day **(C)** are shown for all four digesters in response to perturbation with nalidixic acid, γ-aminobutyric acid (GABA) and sodium phosphate. The fourth reactor acted as a control, with no inhibiting substances. Organic loading rates (OLR) were increased after week 1 (0.43 g/L d^– 1^) and after week 3 (1.29 g/L d^– 1^). At days 0, 56, 70, and 77, 16S-rRNA gene samples were taken for all four reactors (highlighted with horizontal lines in red).

The anaerobic digester experiments lasted 11 weeks and were performed using custom-built continuous stirred tank reactors (CSTRs), which were used in fed-batch configuration. The reactors had a volume of 5 L, with a 3 L working volume (Figure 1). After 1 week without feeding, the reactors received glucose three times a week. For feeding, glucose was dissolved in 150 mL of fresh sludge from a sewage sludge digester. Since feeding events took place discontinuously and the amount of applied substrate and stressors varied during the experiment, the organic loading rate (OLR) could only be estimated. For determining the OLR, the daily flow rate of volatile solids (VS) was calculated by dividing the sum of VS per week by 7. Initially, 1 g L^–1^ of glucose was used, which is equivalent to a loading rate (OLR) of 0.43 gVS L^–1^ d^–1^. After the third week, the reactors received three times a week 3 g of glucose per liter, which corresponds to a loading rate of 1.29 gVS L^–1^ d^–1^ (Figure 2), and this loading rate was retained until the end of the experiment (week 7). Before each feeding event, 150 mL of digestate was removed and used for chemical analysis. Therefore, the retention time was approximately 46.66 days.

Beginning from week 7, three of the four digesters received a chemical stressor with the goal of causing disturbances in the digestion process and the underlying microbiomes (Figure 1). The inhibiting chemicals, which were applied to the different digesters, were fed once a week, using nalidixic acid, GABA and sodium phosphate. The fourth reactor received only the substrate (glucose) and no further supplements. The amount of stressor fed into the respective reactors was increased from 0.5 g L ^–1^ to 5 g L^–1^ for sodium phosphate and from 10 mg L^–1^ to 10 g L^–1^ for nalidixic acid and GABA, as shown in Figure 1. Since both of them are organic substances, adding nalidixic acid and GABA increased the OLR. In weeks 7 and 8, nalidixic acid and GABA were applied in such small amounts that the OLR was only changed to the third decimal place. However, from week 9 onward, much higher amounts of stressors were applied (Figure 1). In week 9, the OLR was increased to 1.43 gVS L^–1^ d^–1^ and during the last 2 weeks, the OLR reached 2.72 gVS L^–1^ d^–1^. In the case of the reactor receiving sodium phosphate, the OLR remained at 1.29 gVS L^–1^ d^–1^ throughout the experiment, since sodium phosphate is an inorganic substance.

### Chemical Analysis

Biogas was analyzed simultaneously with each feeding event (three times a week, Figure 2). The exhaust gas measuring device “Abgasmessgerät VISIT 02 S” from Messtechnik Eheim GmbH (Germany) was used for gas analysis. This measuring device is certified according to the German legal requirements of the Federal Immission Control Act. The device is calibrated at least twice a year with test equipment according to DIN ISO 10012. The detectable gases are oxygen, methane, carbon dioxide, hydrogen sulfide and hydrogen with a volumetric flow rate of 0.8 L min^–1^. Methane and carbon dioxide were detected with an infrared double beam sensor. Oxygen and hydrogen sulfide were detected by a carbon dioxide-compensated electrochemical sensor. The hydrogen content was determined by a palladium sensor. The detection limits for oxygen, carbon dioxide and methane are 0.1 vol%, for hydrogen and hydrogen sulfide 10 ppm with an error of ±1% of the measured value. On analyzing the gas composition, the gas was dried in a custom-built column filled with silica gel. The quantity of the dry gas was analyzed using a common gas meter (BK G6, Elster Handek GmbH Mainz, Germany). Based on the guideline VDI 4630 from the Association of German Engineers (2016), the gas volume was normalized to standard temperature (273 K) and standard pressure (1013 hPa). During the treatment (weeks 7–11), the concentration of chemical oxygen demand (COD) and total volatile fatty acids (TVFAs) were measured once a week. The COD was measured in the untreated sludge (total COD) and in the liquid phase after centrifugation (solubilized COD). The first step of solids separation was carried out via a centrifuge at 13,000 g. The second treatment step was vacuum filtration through a 0.2-μm cellulose-acetate filter (Sartorius AG, Göttingen, Germany). Finally, COD was measured with the Spectroquant COD kit (VWR, Germany) according to the manufacturer’s guidelines. The spectrum of VFAs (lactic acid, formic acid, acetic acid, propionic acid, iso-butyric acid, butyric acid, and valeric acid) was determined by ion chromatography using the Metrosep Organic Acids 250/7.8 column (Model: 882 Compact IC plus, Metrohm AG, Herisau, Switzerland). The applied column is a cation exchange column, which is particularly designed for the determination of VFAs. The mobile phase contained 0.6 mmol L^–1^ of perchloric acid 10 mmol L^–1^ of lithium chloride. The detection limit is 0.25 mg L^–1^. The amount of TVFAs was determined as the sum of all measured VFAs.

### DNA Extraction and Sequencing

Before DNA extraction, samples were washed to reduce the amount of inhibiting substances (especially humic acids). For the first sample (Figure 1, day 0), biomass was sedimented by centrifugation for 5 min at 20,000 *g* and washed several times with sterile phosphate-buffered saline (PBS buffer). Because increasing viscosity sedimentation was impaired in the following extractions, at this point, the centrifugation time was increased to 10 min for all remaining samples. DNA extraction was performed using the DNEasy Power Soil Kit (Qiagen, Netherlands) according to the manufacturer’s instructions. Extracted DNA was quantified using the Qubit dsDNA HS Assay kit (Qubit 2.0 Fluorometer, Thermo Fisher, Waltham, United States). The bacterial full-length 16S rRNA gene was amplified by polymerase chain reaction (PCR) using the following universal primers: S-D-Bact-0008-a-S-16 (5′-AGRGTTYGATYMTGGCTCAG-3′) and S-D-Bact-1492-a-A-16 (5′-TACCTTGTTAYGACTT-3′) (Klindworth et al., 2012). The PCR reaction mix consisted of 200 μM dNTPs, 200 nM of each primer, 1 U of VWR Taq DNA Polymerase (VWR^®^, WR International bvba/sprl, Belgium), 1 x PCR buffer supplemented with MgCl2 (1.5 mM), and 1 ng of DNA template (final volume: 20 μL). The PCR amplification protocol comprised an initial denaturation step at 94°C for 1 min, followed by 35 cycles of amplification (denaturing, 1 min at 95°C; annealing, 1 min at 49°C; extension, 2 min at 72°C) and a final extension at 72°C for 10 min. A negative control (no DNA) was also included. Following the PCR reaction, DNA concentrations were measured using the Qubit dsDNA HS Assay kit (Qubit 2.0 Fluorometer, Thermo Fisher, Waltham, United States). The resulting amplicons were sequenced with Oxford Nanopore MinION, as previously described (Hardegen et al., 2018). In total, 39 samples were multiplexed in the same run using the EXP-PBC096 barcoding kit. The recommended ONT protocols were followed for priming and loading the flow cell. Raw sequences were uploaded at the National Center for Biotechnology Information^1^.

Reads were basecalled with MinKNOW software (core version 3.3.2), and sequencing statistics were assessed by the EPI2ME (v2.59.1896509) ‘Fastq Barcoding’ protocol. Porechop^2^ was applied for detection of the barcodes, demultiplexing of the samples and removal of the adaptors. Finally, reads shorter than 400 base pairs (pb) or with a mean quality below 7 (in PHRED score) were removed.

### Taxonomic Analysis and Modeling

Full-length 16S rRNA sequences generated by MinION were used to obtain a taxonomic profile of each sample. Reads were classified using the Quantitative Insights Into Microbial Ecology (QIIME 1.9.1.) software (Caporaso et al., 2010). OTUs were constructed using the ‘pick_otus.py’ script, and uclust as the picking method (similarity threshold = 97%). Then, ‘pick_rep_set.py’ was run with the default parameters. Taxonomic assignment was carried out with the ‘assign_taxonomy.py’ script, and this consisted of BLAST searches against the latest version (v. 132) of the SILVA database. Finally, ‘make_otu_table.py’ was employed to obtain the final OTU table.

The QIIME results were used to perform simulations based on generalized Lotka–Volterra (gLV) models for each condition studied. The gLV model is an extension of the classic predator-prey Lotka–Volterra model, which allows the prediction of a wider range of relationships (competition, cooperation, neutralism, etc.) among the individual species —or OTUs— coexisting in the same habitat. The interaction could be directly interpreted from the algebraic sign of a coefficient incorporated to the equation (Kuntal et al., 2019). To reduce computation efforts and obtain comparable results, only the most abundant taxa detected in all the experiments were selected for the gLV simulations. Further analyses were performed using the R-software for statistical computing. Differential abundance analyses were carried out using the DESeq2 package (Love et al., 2014; v. 1.18.1) to detect variations in the microbial composition among the different treatments and the control. The ‘phyloseq_to_deseq2’ function was applied to convert the phyloseq object into a DESeq2 object. Then, the DESeq2 main function was applied using the ‘parametric’ option for fitting the dispersion and the ‘Wald test’ option for calculating the significance of the resulting coefficients. The Benjamini–Hochberg method was used for adjusting the *p*-values, and only features with an adjusted *p*-value lower than 0.05 were considered significant.

## Results and Discussion

### Methane Production Upon Addition of Microbial Stressors

The aim of the present work was to cause multiple taxonomic shifts outgoing from the same anaerobic microbiome. Extensive shifts were intended to facilitate the analysis of ecological interactions among involved microorganisms based on population dynamics analysis. Sodium phosphate was used as it is a known stressor in anaerobic digestion processes (Ogata et al., 2016). The antibiotic nalidixic acid was chosen as a stressor, as antibiotics are known to manipulate anaerobic process performance and the involved microbiomes (Mitchell et al., 2013; Mustapha et al., 2016; Bay et al., 2019; Fáberová et al., 2019). GABA was chosen, as high concentrations of butyric acid (an intermediate product from GABA degradation) is known to inhibit syntrophic metabolism in anaerobic digesters (Henson et al., 1985; Zhang et al., 2019).

The experiments started with a low OLR (0.43 gVS L^–1^ d^–1^), with the OLR being elevated after 3 weeks (1.29 gVS L^–1^ d^–1^; Figure 2A), which destabilized the digestion experiments from week 3 until week 6 (Figure 2B). Beginning in week 7, nalidixic acid, GABA and sodium phosphate were also added weekly, and in increasing amounts, to cause a process perturbation, and thus, multiple alterations in the underlying microbiome. Due to the addition of GABA and nalidixic acid, the OLR increased gradually to 2.72 gVS L^–1^ d^–1^ during the last 5 weeks for both cases. In the case of the reactor receiving sodium phosphate, the OLR remained at 1.29 gVS L^–1^ d^–1^ as it is an inorganic substance.

**FIGURE 3:**
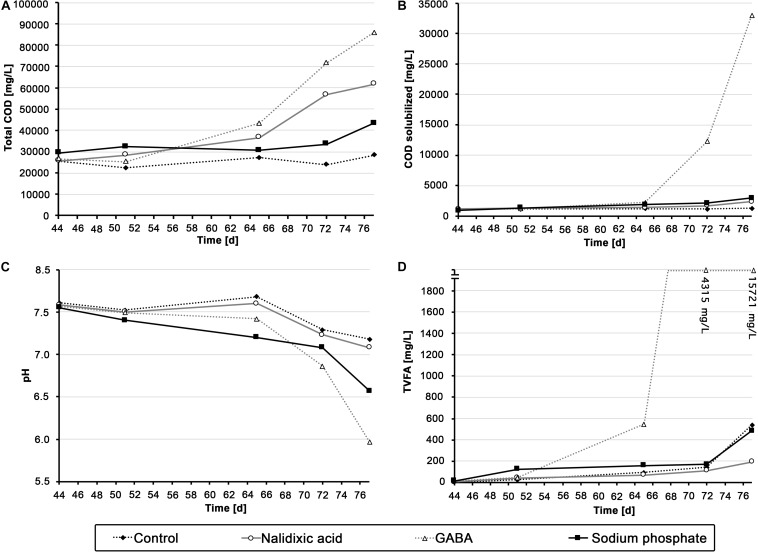
Chemical analysis of the different digesters: Total chemical oxygen demand **(A)**, solubilized chemical oxygen demand **(B)**, pH **(C)** and amount of total volatile fatty acids **(D)** are shown for all the digester experiments, which were perturbed with nalidixic acid, γ-aminobutyric acid (GABA) and sodium phosphate.

During the 11 weeks of the experiment, all reactors received a total of 78.26 g of glucose per liter, which corresponds to a theoretical methane potential of 28.96 L of methane. The control produced 16.66 L of methane per liter of working volume (Figure 2A). Therefore, the digestion efficiency was 57.53%. A similar methane volume would have been expected for the reactor that was supplemented with sodium phosphate, because sodium phosphate cannot be converted into methane. However, the reactor that received sodium phosphate produced only 12.75 L of methane per liter of working volume. Since the cumulative gas volume was already lower than the control, before sodium phosphate was added, a process perturbation due to sodium phosphate cannot entirely explain the lowered cumulative methane volume (Figure 2A). However, the fact that the ratio of methane to total biogas became highly irregular upon the addition of sodium phosphate (Figure 2C, weeks 7 – 11) indicates a process perturbation, which may have affected the methane productivity negatively during the last 5 weeks. This hypothesis is supported by the fact that the pH gradually decreased from 7.55 to 6.57 (Figure 3C).

The reactors receiving nalidixic acid and GABA, in addition to the 78.26 g of glucose, received 21.11 g L^–1^ of the respective stressor. In case of complete degradation, 0.58 L g^–1^ would be expected for nalidixic acid, and 0.49 L g^–1^ for GABA (Supplementary Material S1). In the case of the reactor, which received nalidixic acid, this 21.11 g L^–1^ of stressor corresponds to an additional theoretical methane potential of 12.24 L. In the case of GABA, 21.11 g L^–1^ of stressor corresponds to 10.34 L of methane. Based on the digestion efficiency of 57.53%, which was observed in the control, the reactors receiving nalidixic acid and GABA were expected to produce 7.04 L and 5.95 L more methane per liter than the control did. However, in both cases, the produced volume of methane was extremely close to the control. This suggests that the respective stressors were not entirely converted to methane. One explanation is that the respective stressors were not degradable. Another explanation is an inhibition of the underlying microbiome.

### Chemical Parameters

Although the methane productivity alone did not indicate a very clear variation between the performed digestions experiments, chemical parameters did show some differences. As mentioned above, the ratio of methane to total biogas became highly irregular with the addition of sodium phosphate (Figure 2C, weeks 7-11). From this, one can assume a humble but continuous inhibition of the reactor receiving sodium phosphate, resulting in a pH decrease from 7.55 to 6.57 at the end of the experiment (Figure 3C). Comparing the result of the reactor receiving sodium phosphate to other works, it draws attention that the loading rate must usually be higher to cause acidosis. In an experiment by Goux et al. (2015), where the OLR was gradually increased, acidosis took place approximately at 4 gVS L^–1^ d^–1^. In a recent study by Musa et al. (2018), an UASB reactor showed an even higher stability compared with that of Goux et al. (2015), as the OLR was increased until 15 gCOD L^–1^ d^–1^ before acidosis took place. In the study presented here, the loading rate in the reactor receiving sodium phosphate was based on the works from Goux et al. and Musa et al., and an OLR of 1.29 gVS L^–1^ d^–1^, not close to a range that could cause acidosis. This supports the interpretation that the observed process disturbance was caused by high concentrations of sodium phosphate.

In contrast to the reactor receiving sodium phosphate, a very sudden and heavy shock was observed in the reactor receiving GABA as stressor, which resulted in a strong increase in solubilized COD and TVFAs beginning in week 9 (Figures 3B,D and Supplementary Figure S1). In addition, as expected, this aforementioned COD and TVFA shock coincided with strong irregularities in methane productivity, which was almost fully disrupted by the end of the experiment (Figures 2A,B; day 77) and showed a strongly reduced ratio of methane (Figure 3C, day 77).

Compared with the acidosis events in the aforementioned works from Goux et al. (2015) and Musa et al. (2018), it appears that the OLR in the present study (max. 2.72 gVS L^–1^ d^–1^) was still too small to cause acidosis. As discontinuous fed-batch reactors were used in the present work, one could argue that shock loads may have destabilized the process. However, in an experiment by Nachaiyasit and Stuckey (1997), shock loads with OLR as high as 18 gCOD L^–1^ d^–1^ were applied over a duration of 20 days without causing acidosis. Therefore, it appears unlikely that a substrate overload caused acidosis in the present experiment. A potential explanation could be the aforementioned release of butyric acid, which is a known inhibitor of anaerobic digestion processes (van den Heuvel et al., 1988).

The chemical parameters for the reactor treated with nalidixic acid were particularly unexpected. As explained in the previous section, the methane yield was lower than anticipated, indicating an uncomplete degradation and/or inhibitory effect in the process. Due to the low methane yield, one would expect an increase in TVFAs or COD in the liquid fraction. However, TVFAs and solubilized COD remained at a low level, with a concentration of less than 600 mg L^–1^ (Figures 3B,D). However, at the end of the experiment, a strong increase in the total COD up to 61.60 g L^–1^ was observed. A potential explanation for these findings is an impaired degradation due to adsorption. This hypothesis is supported by the fact that the antibiotics ampicillin, norfloxacin, ciprofloxacin, ofloxacin, tetracycline, roxithromycin, and trimethoprim are mainly removed from sewage systems due to adsorption (Li and Zhang, 2010).

### Taxonomic Profiles After Treatment

**FIGURE 4:**
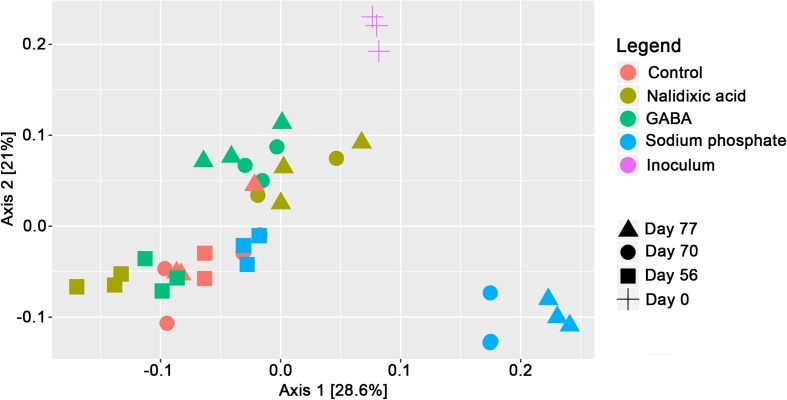
Principal component analysis of 16S-rRNA gene amplicon sequences after calculation of Bray-Curtis dissimilarities at the genus level.

**FIGURE 5:**
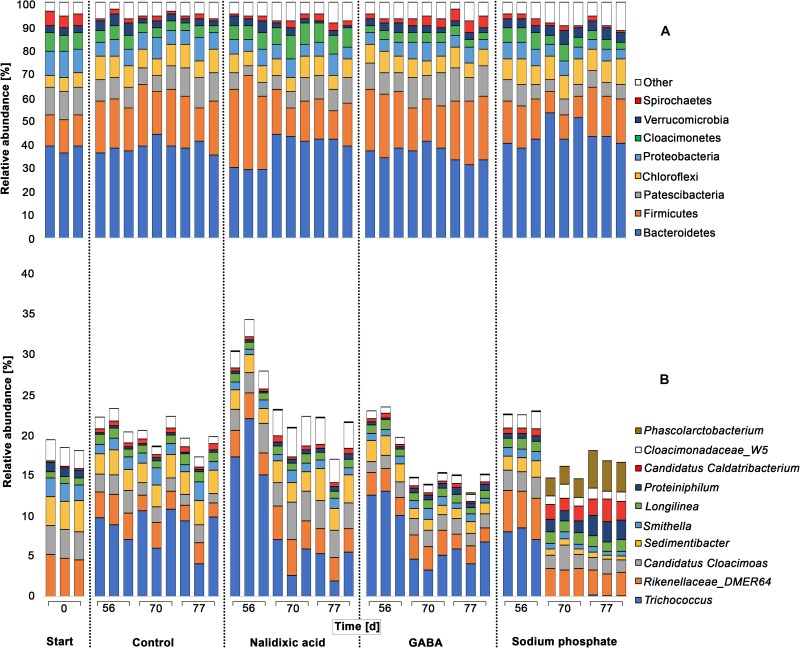
Taxonomic profiles of chemically stressed digester microbiomes: Taxonomic profiles are shown for all experiments (perturbation with nalidixic acid, γ-aminobutyric acid [GABA], sodium phosphate and the control). Results are shown for the 8 most abundant phyla **(A)** and 10 most abundant genera **(B)**. For determining the most abundant phyla and genera, they were sorted after summing up their relative abundancies in all samples. The 16S-rRNA gene amplicons were analyzed for the seed sludge (start) and at three time points during treatment (days 56, 70, and 77). For each timepoint 3 sludge samples were taken and analyzed.

As the basis for population modeling based on the Lotka–Volterra equations, high-throughput sequencing of 16S-rRNA gene amplicons was applied. To create a general overview of the produced data, Bray–Curtis dissimilarities were calculated and analyzed based on a principal component analysis for ordination (Figure 4). The control was extremely different from the rest of the time points. At the beginning of the time period, in which supplementation with the respective chemical stressors started (day 56), all the samples clustered close to each other. However, at day 70, the underlying microbiomes had already clearly diverged. For days 70 and 77, the samples from the reactor receiving sodium phosphate clustered far away from the reactors receiving nalidixic acid and GABA. Interestingly, and despite showing clear differences in the underlying chemical parameters (Figure 3), the reactors receiving nalidixic acid and GABA clustered together. The respective taxonomic profiles for all reactors are shown in Figure 5.

The dominant phyla observed during the experiment were Bacteroidetes (38.82% ± 5.08%) and Firmicutes (19.87% ± 6.68%), which is in line with other studies (Klocke et al., 2007; Sundberg et al., 2013; Abendroth et al., 2015, 2017). Phyla that were observed in minor ratios, were Patescibacteria (9.13% ± 2.73%), Chloroflexi (8.10% ± 1.62%), Proteobacteria (6.56% ± 1.76%), Cloacimonetes (5.36% ± 1.91%), Verrucomicrobia (3.18% ± 1.14) and Spirochaetes (2.41% ± 1.45%). These minor phyla are also typical of digested sewage sludge (Abendroth et al., 2015). The taxonomic patterns were surprisingly similar in all the experiments, despite of the process perturbations due to the addition of nalidixic acid, GABA and sodium phosphate. Such stability at the phylum level has been indicated in other studies. For example, in the work from Calusinska et al. (2018) 20 mesophilic full-scale bioreactors were monitored over a time period of 1 year, and a surprisingly stable core microbiome was revealed. In addition, with harsher conditions, the underlying microbiome shows robustness. For example, the effect of thermoshocks on high-strength liquor from an acidifying pre-treatment stage for an anaerobic digester sludge was investigated, and the frequencies of phyla remained stable despite the harsh heat shocks applied (Abendroth et al., 2018).

Despite of the high robustness of anaerobic digester microbiomes at the phylum level, a shift was detected for Bacteroidetes with addition of nalidixic acid at day 56 (Figure 5A). As the antibiotic nalidixic acid affects gram-negative bacteria, the inhibition of Bacteroidetes was expected. However, more Gram-negative groups should also have been affected. Moreover, the primordial ratio of Bacteroidetes was already re-established at day 70, indicating a rapid adaptation by the involved Gram-negative bacteria. To obtain a deeper understanding of the respective taxonomic shifts, a differential analysis was applied, in which differences among perturbated reactors and the control experiment were analyzed (Supplementary Tables S1–S3). Although the difference for Bacteroidetes at day 56 appeared to be clear compared with days 70 and 77, a differential abundance analysis indicated no significant differences, when comparing the results from day 56 to the control experiment.

In the subsequent discussion, only significant changes with *p* < 0.05 were considered. Compared with the control, it appeared that nalidixic acid caused significant increases in the ratio of Firmicutes, Tenericutes, Cloacimonetes, and Lentispheara. In contrast, Patescibacteria and Nitrospirae showed a significant decrease. Particularly Tenericutes and Nitrospirae seem to have been strongly affected by nalidixic acid, as they were affected at more than one time point. Despite of their statistical significance, it must be highlighted that the respective shifts were extremely small (Figure 5A). An explanation for this robustness may be a high antibiotic resistance of microbiomes from digested sewage sludge, which has already been highlighted by multiple authors (e.g., Amador et al., 2015; Naquin et al., 2015; Karkman et al., 2018; Yin et al., 2019).

Although the performed principal component analysis indicated a high similarity for the microbiomes that were treated with nalidixic acid and GABA (Figure 4), they showed some differences in relation to the control. Atribacteria and Fibrobacteres were reduced in the reactor receiving GABA but not in the reactor receiving nalidixic acid. An increase was observed for the phyla Epsilonbacteraeota and Spirochaetes, which was not observed in the reactor receiving nalidixic acid neither. Interestingly, the phyla Tenericutes and Nitrospirae were also affected by GABA, as was the case with nalidixic acid and with sodium phosphate. This similar shift behavior indicates a high robustness for Tenericutes, as well as a high sensitivity for Nitrospirae. Nitrospirae are known to occur regularly in wastewater treatment plants (Zhang et al., 2017); however, to our knowledge, there are no reports that link Nitrospirae with perturbated conditions in anaerobic digesters. At any rate, the described sensitivity is supported by Daims (2014) work, which highlighted the difficulties in cultivating Nitrospirae, especially the genus Nitrospira. The observed increase for Tenericutes due to the application of all tested stressors is of particular interest, as it is in concordance with a recent work by Braz et al. (2018), where the increase in the abundance of Tenericutes was descrobed as a consequence of an OLR shock.

Other phyla that were significantly impaired due to the application of sodium phosphate were Aegiribacteria, Firmicutes, Proteobacteria, Patescibacteria, and Fibrobacteres. Moreover, there was a significant increase in the ratio of Verrucomicrobia, Synergistetes, Lentisphearae and Atribacteria. Like the reactor receiving nalidixic acid, reactors receiving GABA and sodium phosphate showed only small taxonomic shifts (Figure 5), which again highlights the robustness of the underlying microbiome.

To compare the differences in relative abundancies at the genus level among perturbated reactors and the control experiment (Figure 5B), differential abundance analyses were applied here (Supplementary Tables S4–S6). The most abundant genera, for which significant changes with *p* < 0.05 were observed, were *Trichococcus*, *Sedimentibacter*, *Phascolarctobacterium*, *Cadidatus Caldatribacterium*, and *Proteiniphilum.*

With the addition of nalidixic acid, *Trichococcus* showed a ratio 18.36% ± 2.93% at day 56, which was significantly higher, by 8.80%, than the control. However, no significant differences were detectable at day 70 between the control and the nalidixic acid-receiving reactor anymore, suggesting a fast adaptation. A similar observation was made by Mitchell et al. (2013), where ampicillin with concentrations between 280 and 350 mg L^–1^ inhibited the process only during the early stages. In concordance with this observation, it has recently been described that sewage sludge from wastewater treatment often contains considerable amounts of antibiotic resistance genes (Mengli et al., 2019).

With the addition of sodium phosphate, *Trichococcus* showed a significantly lower ratio than in the control at day 70 Interestingly, *Trichococcus* was not detected in the initial sample (anaerobic digested sludge from a waste water treatment plant). The overall increase of *Trichococcus* at day 56 cannot be explained by the addition of nalidixic acid, GABA or sodium phosphate, as *Trichococcus* was enriched in the control as well.

Like *Trichococcus*, *Sedimentibacter* significantly decreased with the addition of sodium phosphate. At day 56, *Sedimentibacter* showed a ratio of 1.81% ± 0.17% and decreased to a ratio of 0.63% ± 0.09% on days 70 and 77. There were several genera that were significantly enriched upon addition of sodium phosphate in comparison with the control samples, namely, *Phascolarctobacterium*, *Candidatus Caldatribacterium*, and *Proteiniphilum*. At day 56, these three genera showed ratios of 0.02% ± 0.01%, 0.77% ± 0.12 and 0.59% ± 0.09%, respectively. During the last two sampling time points the ratio of these three genera increased to 3.19% ± 1.07%, 2.07% ± 0.46% and 1.96 ± 0.59%.

It should be stressed that the high sensitivity of *Trichococcus and Sedimentibacter*, as well as the increase in relative abundance of *Candidatus Caldatribacterium, Phascolarctobacterium*, and *Proteiniphilum*, is likely linked to phosphate but not conductivity. The highest observed conductivity values for the reactor receiving sodium phosphate was 12.06 mS cm^–1^, but process disturbance due to high conductivity values are usually observed at values higher than 35 mS cm^–1^ (Ogata et al., 2016). By contrast, an inhibitory effect due to high phosphate levels has already been reported at a concentration of 70 mM (Paulo et al., 2005). According to Paulo et al. (2005) the phosphate concentration in the present study reached a level that already inhibited the underlying biocenosis process; in total, approximately 20.5 g L^–1^ was added, corresponding to 125 mM. Other authors have described inhibiting effects due to elevated phosphorous levels too. For example, Sharma and Singh (2001) described phosphate as detrimental for anaerobic sludge granulation during the treatment of distillery effluents. Mancipe-Jiménez et al. (2017) described inhibitory effects due to a sudden increase of phosphorus in the influent during anaerobic liquid waste treatment.

**FIGURE 6:**
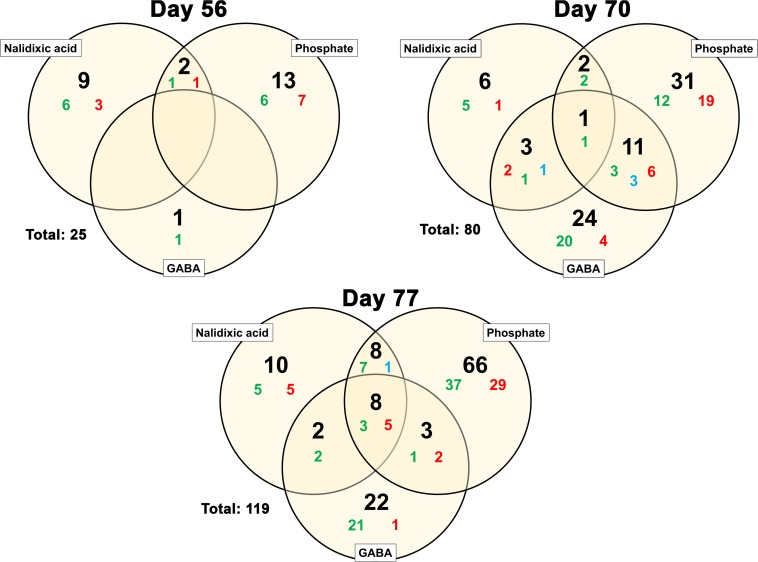
Venn diagrams for genera exhibiting a dynamic behavior in comparison with the control in all reactors: Significant changes in frequency are shown for all the days where DNA samples were analyzed (day 56, 70, and 77). Genera showing an increase in relative abundance are highlighted in green. Genera exhibiting a decrease are highlighted in red. In some cases, the relative abundance of a genus was significantly increased by one stressor but significantly decreased with another stressor. Such cases are highlighted in blue. The sum of all changes (blue, red, and green) is given in black. The total number of OTUs, which were significantly affected in all the reactors is shown to the left of each diagram. Genera from each reactor were compared with the control and only results with a significance of *p* < 0.05 were considered.

From a total of 2995 OTUs, 25 changed their relative abundance on day 56 significantly. On day 70, the number increased to 80 significant changes, which was elevated again on day 77 to 119 significant changes. This number might appear small, but it has to be considered that 2960 OTUs had a relative abundance of less than 1% in the total pool of sequences. To reach a better impression of the severity of the induced stresses at the community level, all significant changes (Supplementary Tables S4–S6) were compared in Venn diagrams. These showed that, with increasing concentrations of stressors, the number of significant taxonomic shifts also increased (Figure 6). From the eight genera that were affected similarly in all three reactors, five showed a significant decrease and three showed a significant increase. The five decreasing genera were *Gracilibacter*, *Geobacter*, *Syntrophobacter*, and two uncultured bacteria. One of these two uncultured bacteria could only be classified on class level (Thermodesulfovibrionia) and the other one on family level (Gracilibacteraceae). The three increasing genera were *Fermentimonas*, *Proteiniphilum* and an uncultured bacterium belonging to the family Acidaminococcaceae.

Comparing the shown taxonomic profiles to the existing literature, it is immediately apparent that the number of works addressing acidosis events on a bacterial level is limited. Many works address acidosis events based only on chemical parameters. Authors of more recent works also address the methanogenic community (e.g., Steinberg and Regan, 2011; Lerm et al., 2012; Tale et al., 2015), but bacterial communities remain underrepresented in most of the works. Among the few works addressing the bacterial community and in relation to the results presented here, an article from Goux et al. (2015) is of particular interest; as in the present study, Goux et al. (2015) observed only small variations at the phylum level. Moreover, the phyla Bacteroidetes, Firmicutes, Chloroflexi, Proteobacteria, Cloacimonetes, Verrucomicrobia and Spirochaetes were also abundant, and on lower taxonomic levels, Goux et al. (2015) described a more intense shift behavior as well. Another work addressing the bacterial community during organic overloading is that of Braz et al. (2019). One of their findings was an increased abundance of fatty acid fermenters and a disturbance of syntrophic bacteria. These two findings are in concordance with the finding presented here of decreased ratios for the genera *Geobacter* and *Syntrophobacter*, which are known syntrophic bacteria (Meher and Ranade, 1993; Liu et al., 2018). The aforementioned increase in *Fermentimonas* and *Proteiniphilum* is also in concordance with the described increase of fatty acid fermenters in the work from Braz et al. (2019). Both *Fermentimonas* and *Proteiniphilum* are known to produce VFAs from a wide range of substrates (Hahnke et al., 2016).

In respect to the observed taxonomic profiles and the detected changes it has to be highlighted that the repeated input of 150 ml of digested sewage sludge during each feeding event might have influenced the results. Invasion of microbial communities is a problem, which has recently been highlighted by Kinnunen et al. (2016). However, the used setting reproduces the normal conditions in the industry and, additionally there are multiple reasons for which it is likely that this had a minor impact on the presented results: The sludge that was used as fed was the same, which was used originally as inoculum. Therefore, the fed did not introduce new kinds of organisms into the system. Moreover, a comparative analysis was performed, in which all the reactors shared the same feeding conditions and, thus, the same “input” microbiota. Therefore, the comparisons are not influenced by this factor. This is supported by a PCA (Figure 4), which shows that the microbiomes diverged and that they were in the end very different from the control.

### Generalized Lotka–Volterra Modeling

To investigate the effect of the different perturbations on the interactions between microorganisms, a gLV was applied. The possibility for fast and robust assessment of microbial interactions directly from microbial time series was recently emphasized by Faust et al. (2018). This model can be used not only to predict the predator-prey interactions in the shape of Lotka–Volterra equations but also to detect a wider range of relationships, including competition, cooperation and neutralism (Kuntal et al., 2019). Based on DNA sequencing, gLV has already been applied various times to investigate microbial interactions in the gut (Weng et al., 2017), in cheese (Mounier et al., 2008), in the coffee-machine bacteriome (Vilanova et al., 2015) or in bacteria grown on pine-tree resin-based medium (Dorado-Morales et al., 2015).

Recently, a graphical user interface (GUI) based interactive platform was published by Kuntal et al. (2019); this is available online^3^, and it automates the estimation of the respective gLV parameters, based on the following equation:

(1) $$\frac{d\,x_{i}}{d\,t}=x_{i}\left(r_{i}+\sum_{j=1}^{n}\propto_{i\,j}x_{j}\right).$$

Here, $\frac{d\,x_{i}}{d\,t}$ corresponds to the rate of growth of species x_*i*_, r_*i*_ represents the intrinsic growth rate and *∝*_*ij*_ is the ‘interaction coefficient’. gLV predictions are based on the algebraic sign of the interaction coefficient. If this coefficient is positive, a beneficial effect is assumed, while prejudicial effects are derived from negative values of the parameter. Finally, if the interaction coefficient is equal to zero, no interaction is assumed between the two taxa.

**FIGURE 7:**
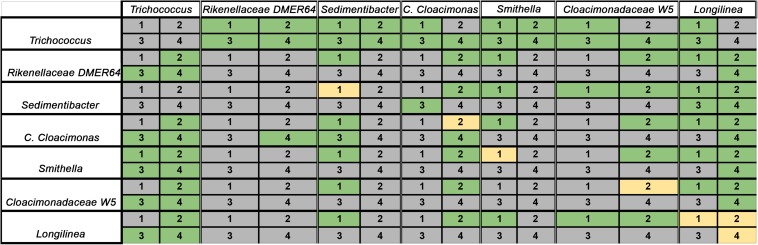
Ecological interactions among the most abundant bacteria in all samples, as deduced from generalized Lotka–Volterra model. Gray: negative interaction; Green: positive interaction; Yellow: no interaction. The numbers 1 – 4 indicate the reactors with the respective stressors: 1: control; 2: perturbation with nalidixic acid; 3: γ-aminobutyric acid (GABA; FG); 4: sodium phosphate (FP).

In the present study, the most abundant bacteria were selected for each condition according to their average relative abundance. Only those OTUs present among the top-10 abundant bacteria in all groups were kept for further Lotka–Volterra modeling (7 OTUs). Applying the gLV on the here presented set of taxonomic data (Figure 7), more positive interactions among the studied taxa were observed in the control experiment (24) than in the rest of the conditions (23 with nalidixic acid, 15 with GABA and 18 with sodium phosphate). In contrast, there were more negative interactions detected in the reactors with nalidixic acid (23), GABA (34) and sodium phosphate (31) than in the control (22). These results suggest that the perturbations introduced in the system tend to create a more competitive environment, in which microorganisms are more likely to interact negatively with each other.

Apart from the total number of microbial interactions (positive, negative, or neutral), it is important to determine which types of pairwise interaction are observed among the taxa in the different set conditions (Figure 7). Interactions involving *Trichococcus* spp. or *DMER64*, in general, were stable in the four conditions. In other words, *Trichococcus* spp. and *DMER64* tend to behave the same way (positively, negatively, or neutrally) with the rest of the studied taxa in all the conditions. However, the pairwise relationships involving other taxa were less homogeneous (i.e., *Cloacimonadaceae W5* negatively interacted with *Trichococcus* in the control experiment, but positive interactions between these two taxa were detected in the rest of the conditions).

Of all the alternative perturbations, the treatment with nalidixic acid proved to be the one with the deepest effects in the interaction patterns compared with the control, whereas the treatments with GABA and sodium phosphate tended to reproduce the same microbial interactions observed in the control (shared interactions of the control with: GABA = 26; sodium phosphate = 27; nalidixic acid = 20). Indeed, the treatment with nalidixic acid displayed a higher number of interactions that were not found in the rest of the conditions (unique interactions in the treatments with nalidixic acid = 11; GABA = 5; sodium phosphate = 0).

Together, our results suggest that antibiotic treatment affects the community interactions present in the anaerobic digesters in a deeper way. Interestingly, all the applied digester conditions resulted in changes in the interaction patterns of the studied microbial taxa. This is of interest in terms of a work from Scherlach and Hertweck (2018), which highlights that microbe–microbe interactions can shape the specific “microenvironment” due to the secretion of chemical mediators. In the context mentioned above, therefore, it would be a promising approach to combine the Lotka–Volterra model (based on 16S-rRNA gene amplicon sequencing) with transcriptomics and metabolomics in future works.

Although the Lotka–Volterra model does not guarantee causality, the high number of genera for which the described correlational behavior was observed suggests that this reflects a biological relationship.

It should be highlighted that the present work was not focused on methanogenic archaea, but rather, it concentrated on bacteria. In the past, the stress responses of methanogenic archaea were extensively investigated using stressors, such as ammonium (Dai et al., 2016), light (Olson et al., 1991), pH and VFAs (Staley et al., 2011). The common view of such works is that, when comparing them to involved bacteria, methanogenic archaea show high sensitivity. Although methanogenic archaea are the most important microorganisms in methane production, since they are performing the final step of anaerobic digestion (methanogenesis), bacteria are key players. Bacteria are responsible for the hydrolysis of complex polymers and the conversion of resulting monomers into hydrogen, acetate, and carbon dioxide, which are the main substrates for methanogenic archaea (Robles et al., 2018). This degradation process involves three phases (hydrolysis, acidogenesis, and acetogenesis). Especially during acidogenesis, various metabolic intermediates are formed; these are of high value for the bio-based industry (Wainaina et al., 2019). The possibility of producing such metabolites during anaerobic digestion also raises the question of how the robustness of the involved bacteria might be overcome in order to manipulate the spectrum of yielded metabolites. In this vein, a recent review article from Strous and Sharp (2018), which explained the importance of ‘designer microbiomes for environmental, energy and health biotechnology,’ can be highlighted.

Results from applying the Lotka–Volterra model for the first time on anaerobic digestion show that microbiomes of anaerobic digesters are not only robust and redundant, but also surprisingly flexible in terms of microbial interactivity. This flexibility indicates that the manipulation of anaerobic microbiomes at the level of microbial interactivity is an ambitious goal that may be achieved more easily with constant digester conditions to prevent the alteration of microbial interaction patterns.

## Conclusion

Emanating from the same microbiome and using different stressors (nalidixic acid, GABA and sodium phosphate), multiple taxonomic shifts were caused for subsequent analysis of populational dynamics. Although the aim of the present work was not to characterize the respective stressors in detail, it can be concluded that sodium phosphate has a particularly strong effect on the bacterial biocenosis, and in contrast, taxonomic profiles were surprisingly stable after addition of nalidixic acid and GABA (in spite of a clear acidosis for the latter case). Taxonomic profiles on phylum level were surprisingly robust. At the genus level, important taxonomic variations were observed especially for the genera *Trichococcus, Candidatus Caldatribacterium, Phascolarctobacterium*, *Proteiniphilum, Gracilibacter*, *Geobacter*, *Syntrophobacter*, and *Fermentimonas*. Therefore, these genera may be promising targets for the surveillance of anaerobic digester microbiomes.

Main objective in the present study was to trigger —and thus shed light— on microbial interactions, based on the gLV model. Except for sodium phosphate, the addition of the respective stressors did not alter taxonomic profiles drastically, indicating a high robustness for the bacterial biocenosis in digested sewage sludge. Interestingly, potential ecological interactions among the key players were strongly affected by all treatments, and in some cases, two pairs of genera showed negative, positive or no correlation, depending on the treatment. Although the presented work suggests a massive resilience and stability of the underlying bacterial biocenosis in respect to the relative abundance of involved bacteria, a highly flexible behavior was observed in terms of microbial interactivity.”

## Data Availability Statement

The datasets generated for this study can be found in the https://www.ncbi.nlm.nih.gov/bioproject/PRJNA554976.

## Author Contributions

BS and CA performed anaerobic digestions experiments. AL-P, CV, and CA performed the taxonomic analyses. BS, CA, AL-P, MP, CV, and CD were writing the manuscript.

## Conflict of Interest

The authors declare that the research was conducted in the absence of any commercial or financial relationships that could be construed as a potential conflict of interest.

## Footnotes

1 https://www.ncbi.nlm.nih.gov/bioproject/PRJNA554976

2 https://github.com/rrwick/Porechop

3 http://web.rniapps.net/webglv

## Supplementary Material

FIGURE S1 Analysis of total volatile fatty acids (TVFAs): The concentrations of formic acid, acetic acid, lactic acid, propionic acid, iso-butyric acid, butyric acid and valeric acid are shown for the control **(A)**, and for the reactions including nalidixic acid **(B)**, GABA **(C)**, and sodium phosphate **(D)**.

TABLE S1 Differential abundance analysis at the phylum level to compare the control and the reactor receiving nalidixic acid: The log2FoldChange of the normalized abundance was calculated using the DESeq2-package (Love et al., 2014). *p*-values of the respective changes were adjusted using the Benjamini-Hochberg method.

TABLE S2 Differential abundance analysis at the phylum level to compare the control and the reactor receiving γ-aminobutyric acid (GABA): The log2FoldChange of the normalized abundance was calculated using the DESeq2-package (Love et al., 2014). *p*-values of the respective changes were adjusted using the Benjamini–Hochberg method.

TABLE S3 Differential abundance analysis at the phylum level to compare the control and the reactor receiving sodium phosphate: The log2FoldChange of the normalized abundance was calculated using the DESeq2-package (Love et al., 2014). *p*-values of the respective changes were adjusted using the Benjamini–Hochberg method.

TABLE S4 Differential abundance analysis at the genus level to compare the control and the reactor receiving nalidixic acid. The log2FoldChange of the normalized abundance was calculated using the DESeq2-package (Love et al., 2014). The *p*-values of the respective changes were adjusted using the Benjamini–Hochberg method.

TABLE S5 Differential abundance analysis at the genus level to compare the control and the reactor receiving γ-aminobutyric acid (GABA). The log2FoldChange of the normalized abundance was calculated using the DESeq2-package (Love et al., 2014). The *p*-values of the respective changes were adjusted using the Benjamini–Hochberg method.

TABLE S6 Differential abundance analysis at the genus level to compare the control and the reactor receiving sodium phosphate. The log2FoldChange of the normalized abundance was calculated using the DESeq2-package (Love et al., 2014). The *p*-values of the respective changes were adjusted using the Benjamini–Hochberg method.

MATERIAL S1 Calculations.
