# Supplementary figures and images for: Chemically Stressed Bacterial Communities in Anaerobic Digesters Exhibit Resilience and Ecological Flexibility
Source: Front Microbiol. 2020 May 12;11:867. doi: 10.3389/fmicb.2020.00867 (PMC7235767; doi:10.3389/fmicb.2020.00867)

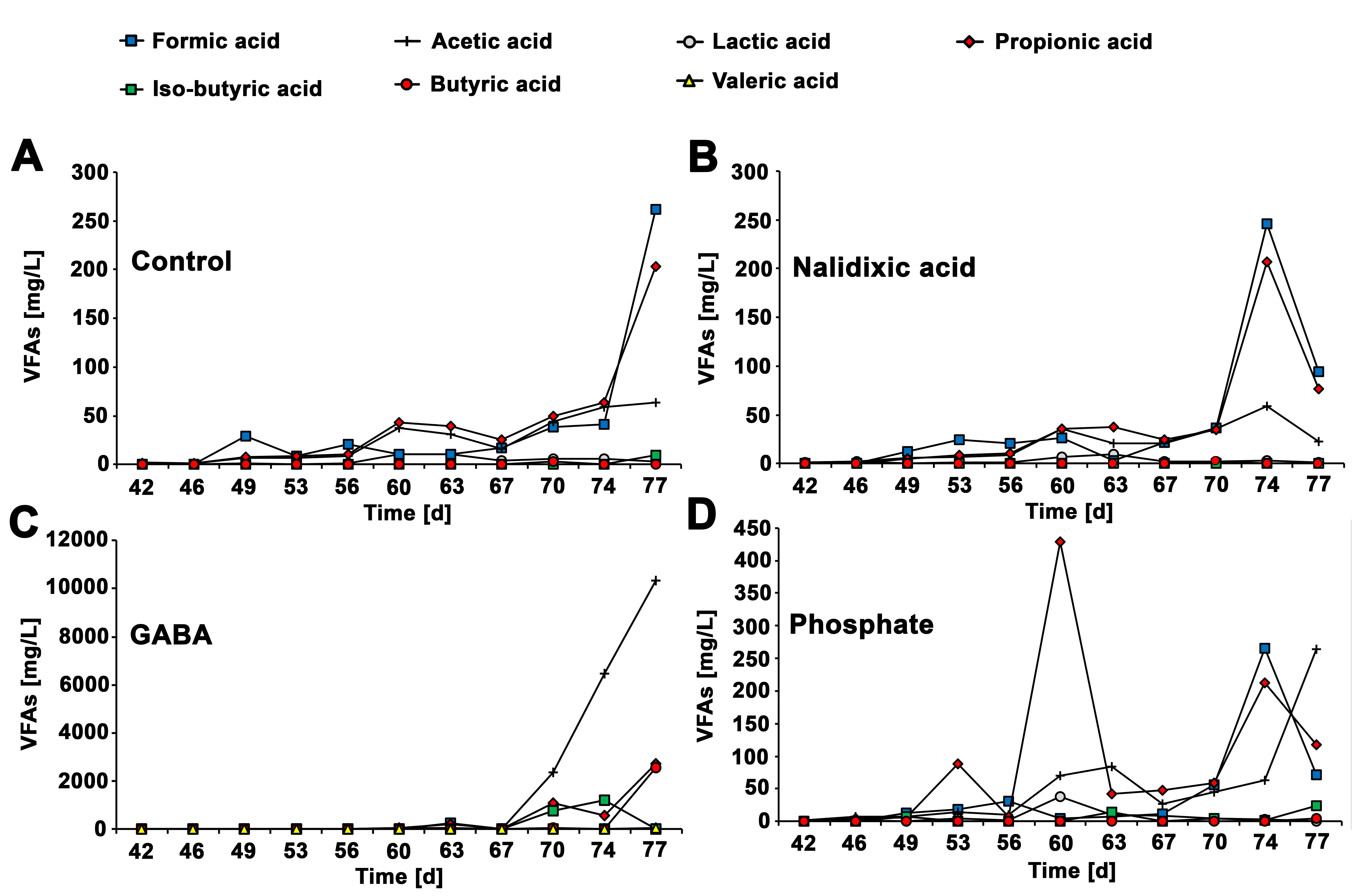

Supplement: FIGURE S1 — Analysis of total volatile fatty acids (TVFAs): The concentrations of formic acid, acetic acid, lactic acid, propionic acid, iso-butyric acid, butyric acid and valeric acid are shown for the control (A), and for the reactions including nalidixic acid (B), GABA (C), and sodium phosphate (D). [file Image_1.TIF]
