# Supplementary material for: Chemically Stressed Bacterial Communities in Anaerobic Digesters Exhibit Resilience and Ecological Flexibility
Source: Front Microbiol. 2020 May 12;11:867. doi: 10.3389/fmicb.2020.00867 (PMC7235767; doi:10.3389/fmicb.2020.00867)
Supplement: TABLE S1 — Differential abundance analysis at the phylum level to compare the control and the reactor receiving nalidixic acid: The log2FoldChange of the normalized abundance was calculated using the DESeq2-package (Love et al., 2014). p-values of the respective changes were adjusted using the Benjamini-Hochberg method. [file Data_Sheet_1.pdf]

**Supplementary Material S1: Calculations**

*Calculation of maximum methane from nalidixic acid and γ-aminobutyric acid (GABA):*

The maximum gas production can be calculated according to Boyle (1976) with the following equation:

$$C_aH_bO_cN_dS_e + \left(a - \frac{b}{4} - \frac{c}{2} + \frac{3d}{4} + \frac{e}{2}\right) \cdot H_2O \rightarrow \left(\frac{a}{2} + \frac{b}{8} - \frac{c}{4} - \frac{3d}{8} - \frac{e}{4}\right) \cdot CH_4 + \left(\frac{a}{2} - \frac{b}{8} + \frac{c}{4} + \frac{3d}{8} + \frac{e}{4}\right) \cdot CO_2 + dNH_3 + eH_2S$$

According to the equation from Boyle, nalidixic acid ($C_{12}H_{12}N_2O_3$) and GABA ($C_4H_9NO_2$) yield maximally 6 mol and 2.25 mol of methane respectively. Multiplying these amounts of methane with 22.414 L (Volume of one mol of an ideal gas) and dividing the resulting volumes by the molar mass of each compound (232.235 g mol$^{-1}$ for nalidixic acid and 103.120 g mol$^{-1}$ for GABA), results in the volume of methane, which can be achieved maximally per g of substance. In the case of nalidixic acid, this results in 0.58 L g$^{-1}$. GABA would yield 0.49 L g$^{-1}$.

*Calculation of the theoretical oxygen demand (thOD) for nalidixic acid*

15 mol of $O_2$ are needed to fully oxidise all carbon and hydrogen atoms in one mol of nalidixic acid ($C_{12}H_{12}N_2O_3$). Subtracting the oxygen, which is already present in nalidixic acid, results in a final oxygen demand of 13.5 mol $O_2$. The contained nitrogen ($N_2$) has an oxygen demand of zero. 13.5 mol $O_2$ correspond to a molar mass of 431.973 g. Dividing this by the molar mass of nalidixic acid (232.235 g mol$^{-1}$) results in a thOD of 1.86 g$O_2$ per gram of nalidixic acid.

**References**

Boyle, W.C (1976). Energy recovery from sanitary landfills - a review. Proceedings of a Seminar Sponsored by the UN Institute for Training and Research (UNITAR) and the Ministry for Research and Technology of the Federal Republic of Germany, Göttingen, 119-138.
