# Supplementary material for: Chemically Stressed Bacterial Communities in Anaerobic Digesters Exhibit Resilience and Ecological Flexibility
Source: Front Microbiol. 2020 May 12;11:867. doi: 10.3389/fmicb.2020.00867 (PMC7235767; doi:10.3389/fmicb.2020.00867)
Supplement: TABLE S2 — Differential abundance analysis at the phylum level to compare the control and the reactor receiving γ-aminobutyric acid (GABA): The log2FoldChange of the normalized abundance was calculated using the DESeq2-package (Love et al., 2014). p-values of the respective changes were adjusted using the Benjamini–Hochberg method. [file Data_Sheet_2.pdf]

Supplementary Table S1: Differential abundance analysis at the phylum level to compare the control and the reactor receiving nalidixic acid: The log2FoldChange of the normalized abundance was calculated using the DESeq2-package (Love et al., 2014). *p*-values of the respective changes were adjusted using the Benjamini-Hochberg method.

Genus	Day 56		Day 70		Day 77	
	**Adjusted p-value**	**log2Fold Change**	**Adjusted p-value**	**log2Fold Change**	**Adjusted p-value**	**log2Fold Change**
Firmicutes	0.0000	1.0020	-	-	-	-
Patescibacteria	0.0210	-0.4636	-	-	-	-
Tenericutes	0.0000	2.5250	0.0000	3.5083	0.0000	4.2877
Cloacimonetes	-	-	0.0156	1.0994	-	-
Nitrospirae	-	-	0.0206	-1.2329	0.0000	-2.6724
Lentisphaerae	-	-	-	-	0.0009	2.3150
