# Supplementary material for: Chemically Stressed Bacterial Communities in Anaerobic Digesters Exhibit Resilience and Ecological Flexibility
Source: Front Microbiol. 2020 May 12;11:867. doi: 10.3389/fmicb.2020.00867 (PMC7235767; doi:10.3389/fmicb.2020.00867)
Supplement: TABLE S3 — Differential abundance analysis at the phylum level to compare the control and the reactor receiving sodium phosphate: The log2FoldChange of the normalized abundance was calculated using the DESeq2-package (Love et al., 2014). p-values of the respective changes were adjusted using the Benjamini–Hochberg method. [file Data_Sheet_3.pdf]

Supplementary Table S2: Differential abundance analysis at the phylum level to compare the control and the reactor receiving γ-aminobutyric acid (GABA): The log2FoldChange of the normalized abundance was calculated using the DESeq2-package (Love et al., 2014). *p*-values of the respective changes were adjusted using the Benjamini-Hochberg method.

**Genus**	**Day 56**		**Day 70**		**Day 77**	
	**Adjusted p-value**	**log2Fold Change**	**Adjusted p-value**	**log2Fold Change**	**Adjusted p-value**	**log2Fold Change**
Epsilonbacteraeota	0.0016	3.4233	0.0000	3.8825	-	-
Nitrospirae	0.0000	1.7690	0.0156	0.8914	0.0001	-1.5715
Atribacteria	-	-	0.0452	-1.0187	0.0000	1.4482
Spirochaetes	-	-	0.0001	1.1573	-	-
Tenericutes	-	-	0.0001	2.0110	0.0000	3.9216
Fibrobacteres	-	-	-	-	0.0236	-3.9080
