# Supplementary material for: Chemically Stressed Bacterial Communities in Anaerobic Digesters Exhibit Resilience and Ecological Flexibility
Source: Front Microbiol. 2020 May 12;11:867. doi: 10.3389/fmicb.2020.00867 (PMC7235767; doi:10.3389/fmicb.2020.00867)
Supplement: TABLE S4 — Differential abundance analysis at the genus level to compare the control and the reactor receiving nalidixic acid. The log2FoldChange of the normalized abundance was calculated using the DESeq2-package (Love et al., 2014). The p-values of the respective changes were adjusted using the Benjamini–Hochberg method. [file Data_Sheet_4.pdf]

Supplementary Table S3: Differential abundance analysis at the phylum level to compare the control and the reactor receiving sodium phosphate: The log2FoldChange of the normalized abundance was calculated using the DESeq2-package (Love et al., 2014). *p*-values of the respective changes were adjusted using the Benjamini-Hochberg method.

**Genus**	**Day 56**		**Day 70**		**Day 77**	
	**Adjusted p-value**	**log2Fold Change**	**Adjusted p-value**	**log2Fold Change**	**Adjusted p-value**	**log2Fold Change**
Aegiribacteri*a*	0.0439	-3.8312	-	-	-	-
Nitrospirae	0.0439	0.6652	-	-	0.0000	-1.8179
Tenericutes	0.0439	1.0165	-	-	-	-
Atribacteria	-	-	0.0001	1.4488	0.0002	1.6077
Firmicutes	-	-	0.0000	-1.3417	-	-
Lentisphaerae	-	-	0.0000	2.9420	0.0000	3.0104
Synergistetes	-	-	0.0390	0.9494	-	-
Verrucomicrobia	-	-	0.0006	0.8892	-	-
Fibrobacteres	-	-	-	-	0.0156	-2.7222
Patescibacteria	-	-	-	-	0.0156	-1.0212
Proteobacteria	-	-	-	-	0.0452	-0.9016
