# Supplementary material for: Chemically Stressed Bacterial Communities in Anaerobic Digesters Exhibit Resilience and Ecological Flexibility
Source: Front Microbiol. 2020 May 12;11:867. doi: 10.3389/fmicb.2020.00867 (PMC7235767; doi:10.3389/fmicb.2020.00867)
Supplement: TABLE S5 — Differential abundance analysis at the genus level to compare the control and the reactor receiving γ-aminobutyric acid (GABA). The log2FoldChange of the normalized abundance was calculated using the DESeq2-package (Love et al., 2014). The p-values of the respective changes were adjusted using the Benjamini–Hochberg method. [file Data_Sheet_5.pdf]

Supplementary Table S4: Differential abundance analysis at the genus level to compare the control and the reactor receiving nalidixic acid. The log2FoldChange of the normalized abundance was calculated using the DESeq2-package (Love et al., 2014). The *p*-values of the respective changes were adjusted using the Benjamini–Hochberg method.

Genus	Day 56		Day 70		Day 77	
	Adjusted p-value	log2Fold Change	Adjusted p-value	log2Fold Change	Adjusted p-value	log2Fold Change
Uncultured bacterium	0.0004	1.8744	0.0003	1.9658	0.0020	1.8543
Uncultured bacterium	0.0000	-0.9829	0.0104	-0.7552	-	-
W5	0.0216	0.6128	0.0000	1.4259	0.0000	1.5104
Ambiguous taxa	0.0010	1.3084	-	-	-	-
Atopococcus	0.0079	1.0624	-	-	-	-
Jeotgalibaca	0.0172	1.2675	-	-	-	-
Trichococcus	0.0002	1.1543	-	-	-	-
Cryptanaerobacter	0.0317	-2.8518	-	-	0.0234	-2.1184
Ambiguous taxa	0.0107	-0.5590	-	-	-	-
Uncultured bacterium	0.0031	-0.7389	-	-	-	-
Acholeplasma	0.0000	2.7021	0.0000	3.9062	0.0000	5.0595
Geobacter	-	-	0.0000	-3.3855	0.0000	-4.2287
Ambiguous taxa	-	-	0.0412	-5.2774	0.0015	-5.8642
Uncultured bacterium	-	-	0.0291	3.2486	-	-
Candidatus Cloacimonas	-	-	0.0027	0.9993	0.0133	1.0293
Uncultured Bacterium	-	-	0.0011	-1.3653	0.0000	-2.7804
Ambiguous taxa	-	-	0.0000	1.7608	0.0000	1.7139
Uncultured bacterium	-	-	0.0104	-0.7552	0.0324	-0.7047
Uncultured bacterium	-	-	0.0000	2.0586	0.0000	2.3520
Paludibacter	-	-	0.0291	2.1530	0.0000	2.6555
Uncultured bacterium	-	-	0.0291	1.9114	0.0002	2.1881
Fermentimonas	-	-	-	-	0.0459	1.9570
Proteiniphilum	-	-	-	-	0.0306	0.9918
Uncultured bacterium	-	-	-	-	0.0133	4.4872
Clostridium sensu stricto	-	-	-	-	0.0000	2.5456
Gracilibacter	-	-	-	-	0.0306	-0.8795
Uncultured bacterium	-	-	-	-	0.0267	-1.0112
Pelotomaculum	-	-	-	-	0.0001	-2.4572
Uncultured bacterium	-	-	-	-	0.0313	1.9719
Uncultured bacterium	-	-	-	-	0.0007	5.8037
Uncultured bacterium	-	-	-	-	0.0012	3.1408
Ambiguous taxa	-	-	-	-	0.0459	-1.9199
Syntrophorhabdus	-	-	-	-	0.0324	0.9406
Desulfuromonas	-	-	-	-	0.0400	-2.6958
Syntrophobacter	-	-	-	-	0.0459	-1.4520
Uncultured bacterium	-	-	-	-	0.0459	2.5434
