# Supplementary material for: Chemically Stressed Bacterial Communities in Anaerobic Digesters Exhibit Resilience and Ecological Flexibility
Source: Front Microbiol. 2020 May 12;11:867. doi: 10.3389/fmicb.2020.00867 (PMC7235767; doi:10.3389/fmicb.2020.00867)
Supplement: TABLE S6 — Differential abundance analysis at the genus level to compare the control and the reactor receiving sodium phosphate. The log2FoldChange of the normalized abundance was calculated using the DESeq2-package (Love et al., 2014). The p-values of the respective changes were adjusted using the Benjamini–Hochberg method. [file Data_Sheet_6.pdf]

Supplementary Table S5: Differential abundance analysis at the genus level to compare the control and the reactor receiving γ-aminobutyric acid (GABA). The log2FoldChange of the normalized abundance was calculated using the DESeq2-package (Love et al., 2014). The p-values of the respective changes were adjusted using the Benjamini–Hochberg method.

Genus	Day 56		Day 70		Day 77	
	Adjusted p-value	log2Fold Change	Adjusted p-value	log2Fold Change	Adjusted p-value	log2Fold Change
Uncultured bacterium	0.0000	1.6322	0.0005	0.9135	0.0038	-1.4536
Candidatus Caldatribacterium	-	-	0.0165	-0.9921	-	-
Fermentimonas	-	-	0.0465	1.8428	0.0015	2.3374
Proteiniphilum	-	-	0.0200	0.8435	0.0004	1.4385
Uncultured bacterium	-	-	0.0465	0.8183	-	-
Uncultured bacterium	-	-	0.0367	-0.3862	-	-
Uncultured bacterium	-	-	0.0126	-4.8641	-	-
Arcobacter	-	-	0.0000	6.2722	-	-
Ambiguous taxa	-	-	0.0106	-1.2965	-	-
Atopococcus	-	-	0.0068	-1.1390	-	-
Jeotgalibaca	-	-	0.0014	-1.7626	-	-
Trichococcus	-	-	0.0004	-1.2632	-	-
Clostridium sensu stricto 7	-	-	0.0000	6.2346	-	-
Sedimentibacter	-	-	0.0000	-1.3011	0.0014	-1.0530
Ambiguous taxa	-	-	0.0000	6.1435	0.0000	6.3342
Anaerovorax	-	-	0.0000	5.8102	0.0000	6.6458
Family XIII AD3011 group	-	-	0.0283	3.6591	0.0000	5.3995
Family XIII UCG-001	-	-	0.0144	4.5974	0.0004	6.1731
[Eubacterium] brachy group	-	-	0.0051	5.4683	0.0005	5.1179
Uncultured bacterium	-	-	0.0000	2.6909	0.0000	4.4816
Gracilibacter	-	-	0.0497	-0.7258	0.0000	-1.8901
Pelotomaculum	-	-	0.0137	1.2433	-	-
Peptoclostridium	-	-	0.0027	4.8964	0.0065	5.4410
Uncultured bacterium	-	-	0.0002	1.4549	-	-
Uncultured bacterium	-	-	0.0344	-2.7173	-	-
Erysipelotrichaceae UCG-004	-	-	0.0002	2.2575	0.0003	2.4622
Uncultured bacterium	-	-	0.0000	8.2684	0.0000	10.0406
Ambiguous taxa	-	-	0.0001	1.5477	-	-
Uncultured bacterium	-	-	0.0137	1.6346	-	-
Uncultured bacterium	-	-	0.0193	1.3389	-	-
Ambiguous taxa	-	-	0.0144	1.1554	-	-
Geobacter	-	-	0.0158	-0.8911	0.0000	-3.4796
Brachymonas	-	-	0.0011	4.0659	0.0054	2.4890
Comamonas	-	-	0.0068	2.8946	-	-
Hydrogenophaga	-	-	0.0002	5.1393	-	-
Pseudomonas	-	-	0.0000	2.3038	-	-
Uncultured bacterium	-	-	0.0000	1.4686	-	-
Thermovirga	-	-	0.0283	-2.9105	-	-
Uncultured bacterium	-	-	0.0011	-1.4872	-	-
Acholeplasma	-	-	0.0002	2.0773	0.0000	4.5100
Uncultured bacterium	-	-	0.0448	2.2467	0.0004	3.3126
Uncultured bacterium	-	-	-	-	0.0242	2.1754
Ambiguous taxa	-	-	-	-	0.0000	-1.5114
Uncultured bacterium	-	-	-	-	0.0000	-1.8320

Bacillus	-	-	-	-	0.0000	2.0920
Uncultured bacterium	-	-	-	-	0.0000	2.0920
Clostridium sensu stricto 15	-	-	-	-	0.0000	7.2804
Clostridium sensu stricto 7	-	-	-	-	0.0000	4.5586
[Eubacterium] nodatum group	-	-	-	-	0.0005	6.1366
Uncultured bacterium	-	-	-	-	0.0000	-1.9991
Intestinimonas	-	-	-	-	0.0041	5.3624
Syntrophomonas	-	-	-	-	0.0000	2.7631
Uncultured bacterium	-	-	-	-	0.0000	8.4611
Acidaminococcus	-	-	-	-	0.0160	5.4893
Phascolarctobacterium	-	-	-	-	0.0000	7.3746
Syntrophobacter	-	-	-	-	0.0001	-1.5503
Termite Treponema cluster	-	-	-	-	0.0004	6.3787
Uncultured bacterium	-	-	-	-	0.0000	1.9973
