# Supplementary material for: Chemically Stressed Bacterial Communities in Anaerobic Digesters Exhibit Resilience and Ecological Flexibility
Source: Front Microbiol. 2020 May 12;11:867. doi: 10.3389/fmicb.2020.00867 (PMC7235767; doi:10.3389/fmicb.2020.00867)
Supplement: MATERIAL S1 — Calculations. [file Data_Sheet_7.pdf]

Supplementary Table S6: Differential abundance analysis at the genus level to compare the control and the reactor receiving sodium phsophate. The log2FoldChange of the normalized abundance was calculated using the DESeq2-package (Love et al., 2014). The *p*-values of the respective changes were adjusted using the Benjamini–Hochberg method.

Genus	Day 56		Day 70		Day 77	
	Adjusted p-value	log2Fold Change	Adjusted p-value	log2Fold Change	Adjusted p-value	log2Fold Change
Brooklawnia	0.0020	-5.0073	-	-	-	-
Ambiguous taxa	0.0193	-3.6208	-	-	-	-
Proteiniphilum	0.0041	0.9713	0.0000	2.1590	0.0000	3.0174
Uncultured bacterium	0.0000	3.5426	0.0000	6.2398	0.0000	7.9401
Uncultured bacterium	0.0017	1.5894	-	-	-	-
Uncultured bacterium	0.0132	-0.2853	-	-	-	-
DMER64	0.0020	0.4625	-	-	-	-
Ambiguous taxa	0.0264	0.9504	0.0286	1.0372	-	-
Sedimentibacter	0.0001	-0.7077	0.0000	-2.1110	0.0000	-2.4219
Gracilibacter	0.0000	-2.4930	0.0000	-3.9376	0.0000	-3.4156
Uncultured bacterium	0.0230	-1.1941	0.0000	-2.6189	0.0000	-3.0912
Uncultured bacterium	0.0040	-2.1204	-	-	0.0000	-2.5690
Geobacter	0.0132	0.8906	-	-	-	-
Acholeplasma	0.0434	1.0484	-	-	-	-
ADurb.Bin063-1	0.0050	-0.5370	-	-	-	-
Candidatus Caldatribacterium	-	-	0.0000	1.5479	0.0000	1.9655
Uncultured bacterium	-	-	0.0157	5.2176	0.0056	4.9762
Fermentimonas	-	-	0.0258	2.2156	0.0000	3.0175
Petrimonas	-	-	0.0008	1.8777	0.0095	1.7869
Paludibacter	-	-	0.0366	1.9661	0.0000	2.8066
Uncultured bacterium	-	-	0.0000	2.0176	0.0000	2.5348
Uncultured bacterium	-	-	0.0106	-5.4860	-	-
Bacillus	-	-	0.0138	-2.1059	0.0279	-1.5279
Aerococcus	-	-	0.0299	-4.7344	0.0129	-3.1970
Ambiguous taxa	-	-	0.0000	-7.1212	0.0000	-5.6883
Atopococcus	-	-	0.0000	-7.8719	0.0000	-4.9720
Carnobacterium	-	-	0.0000	-7.3967	0.0000	-5.7156
Granulicatella	-	-	0.0191	-4.7773	0.0022	-5.5280
Isobaculum	-	-	0.0051	-5.6757	0.0006	-5.9484
Jeotgalibaca	-	-	0.0000	-8.0678	0.0000	-5.7928
Trichococcus	-	-	0.0000	-8.4822	0.0000	-6.0050
Bavariicoccus	-	-	0.0141	-5.2764	0.0362	-4.7049
Enterococcus	-	-	0.0094	-4.4078	-3.7489	0.0012
Butyrivibrio 2	-	-	0.0491	4.7739	-	-
Cryptanaerobacter	-	-	0.0024	1.7291	0.0000	2.7684
Pelotomaculum	-	-	0.0172	1.3465	-	-
Uncultured bacterium	-	-	0.0000	3.6550	0.0000	3.2408
Subdoligranulum	-	-	0.0215	4.6718	0.0000	6.4723
Erysipelothrix	-	-	0.0025	-5.3574	0.0029	-4.4315
Phascolarctobacterium	-	-	0.0000	8.9231	0.0000	12.1850
Uncultured bacterium	-	-	0.0000	3.4124	0.0000	3.8834
Uncultured bacterium	-	-	0.0064	2.6127	0.0022	3.0110
Ambiguous taxa	-	-	0.0491	-1.8001	-	-
Uncultured bacterium	-	-	0.0364	-2.0098	-	-
Ambiguous taxa	-	-	0.0463	-0.6980	0.0000	-1.0084

Uncultured bacterium	-	-	0.0491	-1.3424	0.0000	-1.6414
Uncultured bacterium	-	-	0.0067	-0.9245	0.0000	-1.2871
Syntrophorhabdus	-	-	0.0258	-0.8610	-	-
Halodesulfovibrio	-	-	0.0112	-5.0369	0.0073	-4.1288
Smithella	-	-	0.0001	-1.2613	0.0065	-1.3640
Massilia	-	-	0.0106	-3.9523	0.0029	-3.3306
Pseudomonas	-	-	0.0464	-2.5801	0.0086	-2.8495
RBG-16-49-21	-	-	0.0010	-6.0141	0.0045	-3.6080
Thermovirga	-	-	0.0258	1.8835	-	-
Ambiguous taxa	-	-	0.0002	1.5091	0.0000	2.2072
Uncultured bacterium	-	-	-	-	0.0455	1.3177
Bacteroides	-	-	-	-	0.0012	2.2151
Uncultured bacterium	-	-	-	-	0.0175	2.1195
Uncultured bacterium	-	-	-	-	0.0428	4.7516
Uncultured bacterium	-	-	-	-	0.0123	2.1285
Uncultured bacterium	-	-	-	-	0.0241	-4.3906
Prevotella 1	-	-	-	-	0.0000	7.4858
Prevotellaceae NK3B31 group	-	-	-	-	0.0116	5.5599
Uncultured bacterium	-	-	-	-	0.0011	6.4328
Parabacteroides	-	-	-	-	0.0000	4.8264
Uncultured bacterium	-	-	-	-	0.0086	1.5775
Ambiguous taxa	-	-	-	-	0.0000	-3.1778
Uncultured bacterium	-	-	-	-	0.0000	-2.0834
Ambiguous taxa	-	-	-	-	0.0224	-4.3652
Uncultured bacterium	-	-	-	-	0.0416	-4.6950
Clostridium sensu stricto 12	-	-	-	-	0.0000	2.7175
Ambiguous taxa	-	-	-	-	0.0000	6.3175
Anaerocolumna	-	-	-	-	0.0000	7.6034
Blautia	-	-	-	-	0.0000	8.1394
Hungatella	-	-	-	-	0.0000	11.0296
Incertae Sedis	-	-	-	-	0.0000	6.4360
Lachnoanaerobaculum	-	-	-	-	0.0002	6.3299
Lachnoclostridium	-	-	-	-	0.0000	9.5632
Lachnospiraceae NK4A136 group	-	-	-	-	0.0400	3.2376
Lachnospiraceae UCG-006	-	-	-	-	0.0210	5.0157
Lachnospiraceae UCG-008	-	-	-	-	0.0000	6.2674
[Eubacterium] fissicatena group	-	-	-	-	0.0129	5.2748
[Ruminococcus] torques group	-	-	-	-	0.0007	5.4778
Uncultured bacterium	-	-	-	-	0.0000	8.3244
Anaerofilum	-	-	-	-	0.0000	7.0515
Intestinimonas	-	-	-	-	0.0003	5.8778
Uncultured bacterium	-	-	-	-	0.0317	4.4060
Anaerovibrio	-	-	-	-	0.0002	5.8308
Megasphaera	-	-	-	-	0.0046	5.4309
Ambiguous taxa	-	-	-	-	0.0441	3.2063
Uncultured bacterium	-	-	-	-	0.0000	-1.8561
Uncultured bacterium	-	-	-	-	0.0241	4.0267
Phaselicystis	-	-	-	-	-4.7144	-4.7144
Syntrophobacter	-	-	-	-	0.0093	-2.3028

Uncultured bacterium	-	-	-	-	0.0499	-1.4721
Treponema 2	-	-	-	-	0.0117	1.5472
Uncultured bacterium	-	-	-	-	0.0047	1.0010
Mesotoga	-	-	-	-	0.0244	-0.9552
